# Dynamics of antibody titers to SARS-CoV-2 and clinical outcomes after sotrovimab pre-exposure prophylaxis early after allogeneic hematopoietic stem cell transplantation

**DOI:** 10.1038/s41409-023-01936-2

**Published:** 2023-02-16

**Authors:** Gioele Capoferri, Carla Simone Walti, Pascal Urwyler, Silvio Ragozzino, Jakob R. Passweg, Jörg Halter, Manuel Battegay, Veronika Baettig, Maja Weisser, Brice Arnold, Benedict Morin, Yukino Guetlin, Diana Albertos Torres, Güliz Tuba Barut, Volker Thiel, Adrian Egli, Beatrice Drexler, Nina Khanna

**Affiliations:** 1 Division of Infectious Diseases and Hospital Epidemiology, University Hospital Basel, Basel, Switzerland; 2 Department of Clinical Research, University Hospital of Basel, Basel, Switzerland; 3 Department of Hematology, University Hospital Basel, Basel, Switzerland; 4 Department of Biomedicine, University of Basel, Basel, Switzerland; 5 Institute of Virology and Immunology (IVI), Mittelhäusern and Bern, Bern, Switzerland; 6 Department of Infectious Diseases and Pathobiology, Vetsuisse Faculty, University of Bern, Bern, Switzerland; 7 Division of Clinical Microbiology, University Hospital Basel, Basel, Switzerland

**Keywords:** Infectious diseases, Risk factors

**TO THE EDITOR:**

Recipients of allogeneic hematopoietic cell transplantation (alloHCT) have a higher risk of severe COVID-19 and COVID-19-associated mortality compared to the general population [1, 2]. Vaccination against SARS-CoV-2 remains the most effective intervention to reduce the risk of poor infection outcome [3]. However, in alloHCT, the transplant procedure impairs immune memory to previously administered vaccines and precludes effective vaccine responses early after transplantation [4]. COVID-19 vaccines are therefore recommended earliest three months after alloHCT [5]. To bridge this time between HCT and effective vaccination, pre-exposure prophylaxis (PrEP) for COVID-19 through administration of monoclonal antibodies (mAb) is recommended in several countries since mid-2021 [6, 7]. During the emergence of the Omicron BA.1 variant in winter 2021–2022, only the mAbs sotrovimab and tixagevimab/cilgavimab [8] maintained adequate neutralizing activity in vitro and the PROVENT study provided promising clinical results with tixagevimab/cilgavimab as PrEP [9]. Recent observational studies reported breakthrough infections of 3.8–4.4% and low rates of severe disease in immunocompromised patients receiving PreP with tixagevimab/cilgavimab [10, 11]. However, specific data in newly transplanted alloHCT recipients and correlations to neutralizing antibody responses are lacking. Because tixagevimab/cilgavimab was not available in Switzerland until 04/2022, our institution used sotrovimab as PrEP for COVID-19 in newly transplanted alloHCT recipients. In this prospective observational study, we aimed to generate real-world data on dynamics of neutralization activity and total immunoglobulin (Ig) levels against SARS-CoV-2, safety, and breakthrough infections in the first 3 months after sotrovimab administration in patients who recently underwent alloHCT.

We included alloHCT recipients who received at least one dose of sotrovimab (500 mg intravenously) as PrEP within the first 3 months following alloHCT at the University Hospital Basel from January to March 2022. Patients were routinely followed twice weekly in the outpatient clinic. SARS-CoV-2 PCR from respiratory samples was performed on day 30 and 60 after sotrovimab administration as well as in the event of symptoms. Blood samples were collected on day 0 before and on days 7, 30 and 60 after administration. Patient data were collected until day 90. Results were censored earlier for SARS-CoV-2 vaccination or infection or for administration of a second sotrovimab infusion. Anti-Spike/receptor binding domain (anti-S/RBD) total Ig levels were measured with a semi-quantitative Elecsys® Anti-SARS-CoV-2 immunoassay (Roche). Neutralizing antibody titers were measured with a microneutralization assay using wild-type Wuhan, Delta and BA.1 viruses and spike BA.2 pseudotype virus. Full methods are described in the Supplemental Methods. The study was approved by Ethics Committee Northwest and Central Switzerland (Project-ID 2020-00769).

Of the 41 patients who underwent alloHCT from 11/2021 to 03/2022, 36 (88%) were included into the study. Five patients were not included because of death (*n* = 1) or transfer to another hospital (*n* = 2) prior to sotrovimab administration, or patients wish not to receive sotrovimab (*n* = 2).

The 36 included patients received sotrovimab at a median of 29 days (range 17–80) after alloHCT. Prior to HCT, 26 (72%) patients had received at least one dose of vaccination against SARS-CoV-2, 7 (19%) patients had a prior SARS-CoV-2 infection, and only 6 (17%) patients neither had prior vaccination nor infection. No patient was lost to follow-up. Baseline characteristics are in Supplemental Table 1.

Before sotrovimab administration, the median level of anti-S/RBD total Ig was 1016.5 U/ml (range, 45.2–2500), 8 (22.2%) patients had absent or very low levels (<256 U/ml). Only 7 (19.4%) had levels above the upper limit of detection. After sotrovimab administration, anti-S/RBD total Ig increased and remained stable until day 60 with levels above the upper limit of detection in 31 (86.1%) patients (Fig. 1a).

**Fig. 1:**
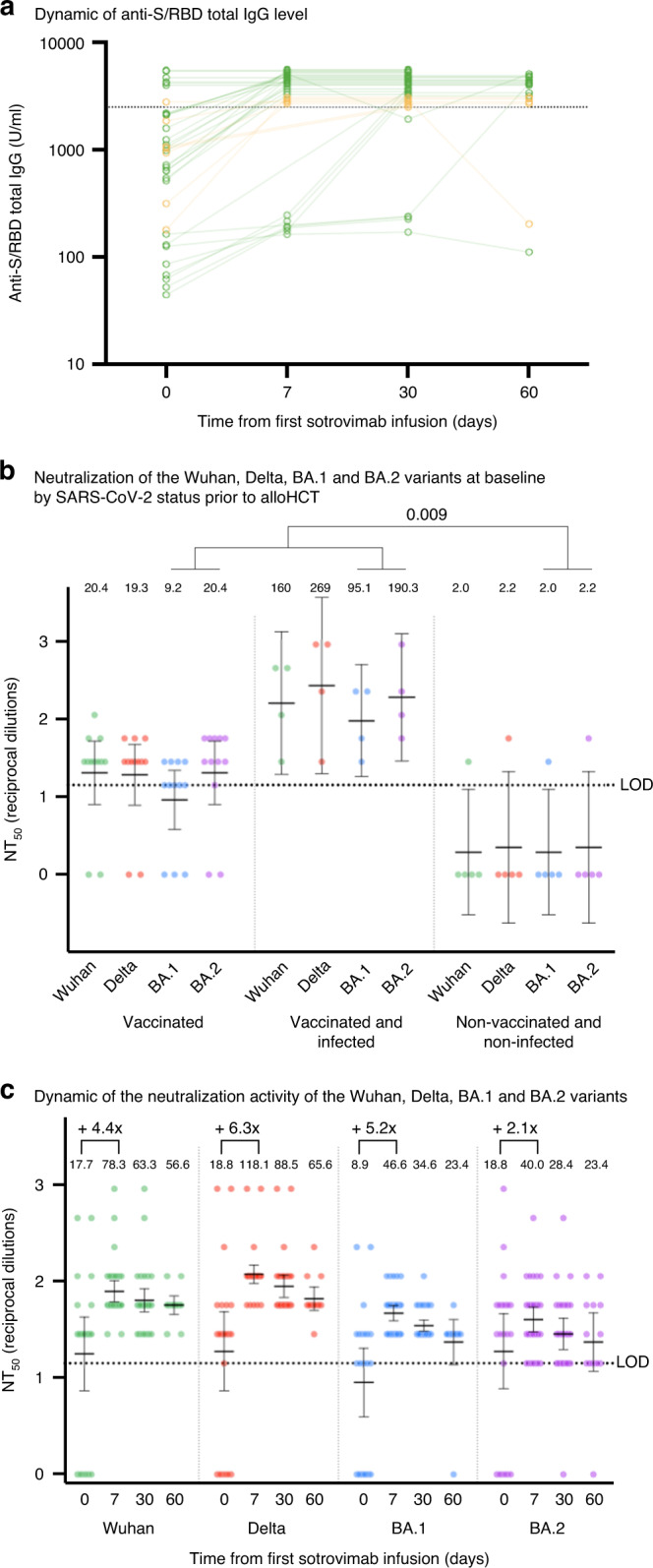
Dynamics of total immunoglobulin levels and neutralization activity against SARS-CoV-2. **a** Spaghetti plot showing the dynamics of anti-S/RBD total Ig levels (logarithmic scale) on day 0 before and on day 7, 30 and 60 after sotrovimab administration. Green dots indicate individual samples of patients without breakthrough SARS-CoV-2 infection; yellow dots indicate individual samples of patients with breakthrough infection. Lines connect results from individual patients. Values above the upper limit of detection (> 2500 U/ml) are presented scattered in order to allow a graphical representation without excessive overlap. Included are 3 patients who received a second dose of sotrovimab on day 30 because of persistent low level of anti-S/RBD total Ig. **b**, **c** Neutralizing antibody titers at baseline (before administration of sotrovimab) sorted by SARS-CoV-2 status pre-transplant and dynamic after pre-exposure prophylaxis with sotrovimab early after alloHCT. 50% neutralization titers (NT_50_) are presented on a logarithmic scale. Dots indicate individual samples. The horizontal dotted line along the x axis indicates the limit of detection (LOD), which was set at the serum dilution of 1:10 (NT_50_, 14.142). The geometric mean values for the NT_50_ are presented graphically (horizontal black lines) and shown as absolute numbers at the top of the plots.  bars represent 95% confidence intervals.

Before sotrovimab administration, 6 (19.4%) patients had no detectable neutralizing antibodies. SARS-CoV-2 vaccination and infection prior to alloHCT were associated with a higher neutralizing activity against BA.1 and BA.2 after alloHCT, but before sotrovimab administration (*p* = 0.009) (Fig. 1b).

After sotrovimab administration, the geometric mean of neutralizing antibody titers across all patients increased and peaked on day 7 with a mean fold increase from baseline of 4.4 for Wuhan, 6.3 for Delta, 5.2 for BA.1 and 2.1 for BA.2 (Fig. 1c). Neutralizing antibodies against BA.1 were still detectable in 31/31 (100%) patients at day 30 and in 13/14 (92.9%) patients at day 60, and against BA.2 in 30/31 (96.8%) patients and in 13/14 (92.9%) patients, respectively. However, there was a lower activity of sotrovimab against BA.1 and especially BA.2, as previously shown in in vitro studies [8]. Before sotrovimab administration, the anti-S/RBD total Ig and the neutralizing antibody titers showed a strong correlation (data not shown). After sotrovimab administration, most anti-S/RBD total Ig levels were above the upper limit of detection, and thus, the correlation could not be assessed.

Only one adverse event involving transient fever and rash was reported in one patient (2.8%) after sotrovimab administration. All patients were alive at day 90 after sotrovimab administration.

Breakthrough infection with SARS-CoV-2 occurred in 7 patients (19.4%), at the median 42 days (range 15–81) after sotrovimab administration. Four patients were asymptomatic and tested positive at day 30 or 60 in the routine PCRs. Three patients had a mild course; two were hospitalized for observation for 1 and 4 days, respectively, but none required supplemental oxygen. SARS-CoV-2 sequencing was performed in 3 cases and BA.1 and BA.2 were detected in 1 and 2 patients, respectively. Patients with breakthrough infections had similar neutralizing activity against BA.1 and BA.2 (data not shown) and similar clinical baseline characteristics compared to patients without breakthrough infection (Supplemental table 1) with the exception of the donor source, which was more often a mismatched unrelated donor (5 [71%] vs. 7 [24%]).

Samples from four patients collected after breakthrough infection showed an increase in neutralizing antibodies, suggesting that endogenous antibody production can develop early after alloHCT despite severe immunosuppression.

The strengths of this study are the real-world and longitudinal design, the inclusion of a cohort that is both, extremely vulnerable to infections and underrepresented in studies on COVID-19 prevention, the consecutive inclusion of all alloHCT recipients who received sotrovimab during the omicron wave in early 2022, and the use of wild-type viruses for the microneutralization assays (except for BA.2). Our study has limitations including the single-centre design, the relatively small patient number, the lack of information regarding the SARS-CoV-2 HCT donor status, and the absence of a control group which limits conclusions on efficacy. Testing was restricted to humoral immunity as this is influenced by sotrovimab, however, cellular responses may be even important, especially in breakthrough infections.

In conclusion, our data demonstrate that patients with prior SARS-CoV2 vaccination or infection were more likely to have detectable neutralizing antibodies early after alloHCT. Administration of a PrEP with sotrovimab within the first 3 months after alloHCT was safe and resulted in relevant increase in circulating anti-S/RBD total Ig and effective neutralizing activity against all viral strains. Despite detectable neutralizing activity, SARS-CoV-2 breakthrough infection occurred in 19.4% of patients, however, it is reassuring that no patient had a severe course. It remains unclear whether the high rate of breakthrough infections was due to limited efficacy of sotrovimab against the circulating BA.1 and BA.2 variants or the high omicron surge in Switzerland, which affected approximately 10% of the Swiss population per week during the study period. Efficacy data on PrEP adjusted for currently circulating variants and randomized trials in this population are needed.

## Supplementary information

Supplementary content

## Data Availability

GC, CSW and NK had full access to all study data (available upon data-specific request).
