# Supplementary material for: Dynamics of antibody titers to SARS-CoV-2 and clinical outcomes after sotrovimab pre-exposure prophylaxis early after allogeneic hematopoietic stem cell transplantation
Source: Bone Marrow Transplant. 2023 Feb 16;58(5):597–9. doi: 10.1038/s41409-023-01936-2 (PMC9933011; doi:10.1038/s41409-023-01936-2)
Supplement: Supplementary file 1 — Supplementary content [file 41409_2023_1936_MOESM1_ESM.docx]

**Supplementary Content**

**Supplemental Methods**

Anti-Spike/receptor binding domain (Anti-S/RBD) immunoassay

Anti-S/RBD total Immunoglobulin (Ig) levels were measured with a semi-quantitative Elecsys® Anti-SARS-CoV-2 immunoassay (Roche). According to the manufacturer’s instructions, the lower limit of detection of anti-S/RBD total Ig was 0.4 U/ml, the upper limit was 2500 U/ml, and values above 0.8 U/ml were considered positive.

Microneutralization assay

Neutralizing antibody titers were measured with a microneutralization assay using wild-type Wuhan, Delta and BA.1 viruses and spike BA.2 pseudotype virus (testing was done at the Institute of Virology and Immunology, Thiel Lab, Bern, Switzerland), and reported as 50% neutralization titers (NT_50_). NT50 were calculated with Spearman–Kärber formula.

Statistical analysis

The data analysis was performed with Stata. The correlation between baseline characteristics and neutralizing activity against BA.1 and BA.2 before (on day 0) and after (on day 30) administration of sotrovimab was assessed using a Fisher’s exact test. The comparison of baseline characteristics between patients with and without SARS-CoV-2 breakthrough infection was performed using a Fisher’s exact test as well.

**Supplemental Table 1**

Baseline characteristics and clinical outcomes of all patients and by SARS-CoV-2 breakthrough infection after sotrovimab administration.

| **Baseline characteristics** | **All patients**  **(N=36)** | **No breakthrough infection**  **(N=29)** | **Breakthrough infection  (N=7)** |
| --- | --- | --- | --- |
| Age, median (range) - years | 58 (21 – 73) | 59 (21 – 73) | 49 (36 – 65) |
| Female sex | 15 (41.7) | 10 (34.5) | 5 (71.4) |
| HCT indication |  |  |  |
| Acute lymphocytic leukemia | 3 (8.3) | 2 (6.9) | 1 (14.3) |
| Acute myeloblastic leukemia | 16 (43.4) | 12 (41.4) | 4 (57.1) |
| Myelodysplastic syndrome | 7 (19.4) | 6 (20.7) | 1 (14.3) |
| Lymphoma | 2 (5.6) | 1 (3.5) | 1 (14.3) |
| Myeloproliferative neoplasm | 3 (8.3) | 3 (10.3) | 0 |
| Other | 5 (13.9) | 5 (17.2) | 0 |
| Best response of the underlying malignancy prior to alloHCT |  |  |  |
| Complete response | 23 (63.9) | 18 (62.1) | 5 (71.4) |
| Partial response | 0 | 0 | 0 |
| Stable disease | 13 (36.1) | 11 (37.9) | 2 (28.6) |
| Progressive disease | 0 | 0 | 0 |
| HCT graft source |  |  |  |
| Bone marrow | 3 (8.3) | 3 (10.3) | 0 |
| Peripheral blood stem cells | 33 (91.7) | 26 (89.7) | 7 (100) |
| HCT donor |  |  |  |
| Matched related donor | 10 (27.8) | 10 (34.5) | 0 |
| Mismatched related donor | 1 (2.8) | 1 (3.5) | 0 |
| Matched unrelated donor | 10 (27.8) | 8 (27.6) | 2 (28.6) |
| Mismatched unrelated donor | 12 (33.3) | 7 (24.1) | 5 (71.4) |
| Haploidentical donor | 3 (8.1) | 3 (10.3) | 0 |
| Conditioning regime |  |  |  |
| Myeloablative conditioning regimen | 8 (22.2) | 6 (20.7) | 2 (28.6) |
| Intermediate intensity conditioning regimen | 10 (27.8) | 8 (27.6) | 2 (28.6) |
| Reduced intensity conditioning regimen | 18 (50.0) | 15 (51.7) | 3 (42.8) |
| T cell depletion |  |  |  |
| None | 3 (8.3) | 3 (10.3) | 0 |
| Anti-thymocyte globulin | 28 (77.8) | 22 (75.9) | 6 (85.7) |
| Other | 5 (13.9) | 4 (13.8) | 1 (14.3) |
| B cell depletion within 6 months prior to alloHCT | 4 (11.1) | 4 (13.8) | 7 (100) |
| COVID-19 before alloHCT | 7 (19.4) | 5 (17.2) | 2 (28.6) |
| COVID-19 vaccination before alloHCT | 26 (72.2) | 21 (72.4) | 5 (71.4) |
| No COVID-19 or COVID-19 vaccination before alloHCT | 6 (16.7) | 4 (13.8) | 2 (28.6) |
| Time from HCT to baseline Ig measurement, median (range) - days | 29 (16 – 78) | 32 (16 – 78) | 21 (18 – 78) |
| Anti-S/RBD total Ig level at baseline, median (range) - U/ml | 1016.5 (45.2 – 2500) | 992 (45.2 – 2500) | 1041 (182 – 2500) |
| Anti-N total Ig level at baseline, median (range) - U/ml | 2.8 (0.2 – 25.4) | 2.8 (0.2 – 25.4) | 2.3 (0.8 – 6.7) |
| NT_50_ against BA.1 at baseline, geometric mean | 8.9 | 7.7 | 17.3 |
| NT_50_ against BA.2 at baseline, geometric mean | 18.8 | 17.7 | 24.5 |
| SARS-CoV-2 PCR negative at baseline | 36 (100) | 29 (100) | 7 (100) |
| Prednisone >20 mg/d or equivalents for at least 5 consecutive days* | 20 (55.6) | 17 (58.6) | 3 (42.9) |
| **Clinical outcomes** |  |  |  |
| COVID-19 breakthrough infection | 7 (19.4) | - | - |
| Hospitalization due to COVID-19 | 2 (5.6) | - | - |
| ICU admission or death due to COVID-19 | 0 | - | - |
| Acute graft-versus-host disease† | 23 (63.9) | 20 (69) | 3 (42.9) |
| Hospitalisation due to causes unrelated to COVID-19 | 4 (11.1) | 4 (13.8) | 0 |

Data presented as n (%) if not indicated otherwise. NT_50_ indicates 50% neutralization titer; Anti-S/RBD total Ig, anti-Spike/receptor binding domain total immunoglobulin; Anti-N total Ig, Anti-Nucleocapsid total Immunoglobulin.

*Administered any time after alloHCT until 90 days after the last sotrovimab infusion.

† Any time from alloHCT until 90 days after the last sotrovimab infusion.
